# Dyslipidaemia in a Black African diabetic population: burden, pattern and predictors

**DOI:** 10.1186/s13104-017-2916-y

**Published:** 2017-11-09

**Authors:** William Lumu, Leaticia Kampiire, George Patrick Akabwai, Richard Ssekitoleko, Daniel Ssekikubo Kiggundu, Davis Kibirige

**Affiliations:** 1 Department of Medicine and Diabetes/Endocrine Unit, Mengo Hospital, Kampala, Uganda; 2 Infectious Diseases Research Collaboration, Kampala, Uganda; 3 Baylor College of Medicine Children’s Foundation, Kampala, Uganda; 4 Infectious Disease Unit, Mulago National Referral and Teaching Hospital, Kampala, Uganda; 5 Nephrology Unit, Mulago National Referral and Teaching Hospital, Kampala, Uganda; 6 Department of Medicine, Uganda Martyrs Hospital Lubaga, P.O. BOX 7146, Kampala, Uganda

**Keywords:** Dyslipidaemia, Burden, Predictors, Adult diabetics, Uganda

## Abstract

**Objectives:**

This study sought to assess the burden, pattern and predictors of dyslipidaemia in 425 adult diabetic patients in Uganda.

**Results:**

The median (IQR) age of the study participants was 53 (43.5–62) years with a female majority (283, 66.9%). Dyslipidaemia defined as presence of ≥ 1 lipid abnormalities was observed in 374 (88%) study participants. Collectively, the predictors of dyslipidaemia were: female gender, study site (private hospitals), type of diabetes (type 2 diabetes mellitus), statin therapy, increased body mass index and diastolic blood pressure. Proactive screening of dyslipidaemia and its optimal management using lipid lowering therapy should be emphasised among adult diabetic patients in Uganda.

**Electronic supplementary material:**

The online version of this article (10.1186/s13104-017-2916-y) contains supplementary material, which is available to authorized users.

## Introduction

Globally, cardiovascular diseases (CVD) account for the greatest adult morbidity and mortality. According to the 2012 World Health Organisation estimates, about 17.5 million people died from CVD. This was equivalent to 31% of all global deaths and the majority (about 80%) of these deaths occurred in low and middle income countries [1]. Diabetes mellitus (DM) is a recognised coronary artery disease equivalent which accounts for about 75% of atherosclerotic related mortality in diabetic patients [2]. Diabetic dyslipidaemia is defined by a high plasma TGL concentration, low HDL cholesterol concentration and increased concentration of small dense LDL-cholesterol particles [3].

Despite compelling evidence that dyslipidaemia is highly prevalent among patients with type 2 diabetes mellitus (T2DM), there are few published studies about diabetes-dyslipidaemia co-morbidity in Uganda [4–6]. These available studies have limitations like: small sample sizes, being single hospital based, the varying study definitions of dyslipidaemia and did not investigate the independent predictors of dyslipidaemia.

This study investigated the burden, pattern and predictors of dyslipidaemia in Uganda.

## Main text

### Methods

This analytical cross sectional study was performed from 1st September 2014 to 31st July 2015 at outpatient diabetic clinics of 3 urban tertiary hospitals in Kampala, Uganda. These hospitals serve an urban population of approximately 2 million people. The outpatient diabetes clinics in these hospitals function only once a week and an average of 35 patients are reviewed by either a general practitioner or specialist physician. Comprehensive diabetic education, body mass index (BMI), blood pressure (BP) and fasting blood sugar measurement are regularly done at every clinical review.

The patients that were eligible for enrolment in the study were those aged ≥ 18 years with a confirmed diagnosis of diabetes using either fasting blood glucose levels, an oral glucose tolerance test, HbA1c or random blood sugar level in the presence of symptoms of diabetes, had been receiving treatment at the study centre for a minimum of 6 months and had provided informed consent. These were enrolled consecutively until the desired sample study size was reached.

All critically ill patients that required intensive care in-patient management were excluded from the study.

### Sample size calculation

Basing on one of the objectives of the study i.e. to determine the burden of dyslipidaemia, the prevalence (P) of low high dense lipoprotein cholesterol (HDLC) of ≤ 40 mmol/l of 52% as reported in the study by Kamara et al. among 150 adult diabetic patients in Southern Western Uganda was used as the prevalence of dyslipidaemia [5]. Using the formula: n = Z^2^P (1 − P)/d^2^ where Z (normal value corresponding to the 95% confidence interval) = 1.96, P = 0.52 and d = 0.05, a sample size of 383 adult diabetic patients was obtained. However, a total of 425 adult patients were enrolled.

### Data collection

Using a pre tested questionnaire, information about the study participants’ socio-demographic characteristics, co-morbidities, type of diabetes, age at diagnosis of DM, duration since diagnosis and drug history was collected by the trained study team. All study participants had their BP, height and weight (for BMI calculation) measured. These obtained study variables are known to be associated with dyslipidaemia in clinical studies and literature.

A venous blood sample was withdrawn from each patient after providing informed consent by the study phlebotomist for analysis of the glycated haemoglobin (HbA1c), low density lipoprotein cholesterol (LDLC), HDLC, triglyceride (TGL) and total cholesterol (TC) concentrations using a full automated COBAS^®^ integra 400 (Roche Diagnostics GmbH) machine at each participating hospital.

### Statistical analysis

The collected study information was entered into Microsoft Excel data base and analysed using Stata software version 12.1. The patient characteristics of interest were reported as frequency and percentage for categorical variables and median and inter-quartile range (IQR) for continuous variables which were not normally distributed.

Dyslipidaemia was defined as presence of ≥ 1 lipid abnormality among the study participants. The following lipid concentrations were considered abnormal as according to the 2015 American Diabetes Association standards of care of diabetes [7] and the 2014 National Lipid Association annual summary of clinical lipidology summary on patient-centred evaluation, management and care of patients with dyslipidaemia [8]: LDLC > 2.6 mmol/l, HDLC < 1.3 mmol/l, TGL > 1.7 mmol/l, TC > 5 mmol/l and non HDLC < 3.4 mmol/l. Non HDLC, an integral lipid parameter in lipidology was calculated using the formula: non HDLC = TC-HDLC in mmol/l [8]. Frequencies of patients with abnormal concentrations for each lipid parameter and those with ≥ 1 lipid abnormality were calculated to determine the burden and pattern of dyslipidaemia. To determine associations between the study variables of interest and the 3 abnormal lipid parameters of interest i.e. elevated LDLC, TGL and non NDLC, bivariate analyses using Chi square test was performed. All variables with a p value of < 0.2 were considered significant at bivariate analysis. Multivariate analysis using logistic regression was then performed to identify the independent predictors. A *p* value of < 0.05 and confidence intervals not including 1 were considered to be statistically significant.

## Results

### Socio-demographic and clinical characteristics

The median age of the study participants was 53 (43.5–62) years. Females constituted the greatest proportion of study participants (284, 66.9%) and hypertension co-morbidity was reported in 292 (68.9%) study participants (summarised in Table 1).

**Table 1:** Socio-demographic and clinical characteristics of the study participants (N = 425)

Variable	N (%)
Age in years, median (IQR)	53 (43.5–62)
Gender, n (%)	
Male	140 (33.02)
Female	284 (66.98)
Education level, n (%)	
None	38 (8.96)
Primary	165 (38.92)
Secondary	141 (33.25)
Tertiary	79 (18.63)
Occupation, n (%)	
Employed	212 (50)
Unemployed	212 (50)
Marital status, n (%)	
Married	259 (61.08)
Cohabiting	10 (2.36)
Single	47 (11.08)
Divorced	41 (9.67)
Widow/widowed	67 (15.80)
Place of residence	
Rural	136 (32.08)
Urban	288 (67.92)
Study site	
Government	199 (46.82)
Private	226 (53.18)
Smoking	
Yes	10 (2.35)
No	415 (97.65)
Known HT	
Yes	292 (68.87)
No	132 (31.13)
HIV co-existent	
Yes	17 (4.00)
No	408 (96.00)
FH-DM	
Yes	264 (62.26)
No	160 (37.74)
Type of DM	
Type 1 DM	55 (13.13)
Type 2 DM	364 (86.87)
Drug history	
Diet alone	3 (0.71)
Metformin alone	79 (18.59)
Met + SU	127 (29.88)
Met + SU + TZD	16 (3.76)
Met + incretins	8 (1.88)
Insulin alone/+ met	188 (44.34)
Statins	89 (20.94)

Variable	Median (IQR), N = 425
Age at diagnosis in years	47 (37–55)
Duration with DM in years	4.5 (2–10)
BMI in kg/m^2^	27 (23–30.6)
HbA1c (%)	9 (6.8–12.4)
LDLC in mmol/l	2.9 (2.3–3.84)
HDLC in mmol/l	1.19 (0.9–1.42)
TC in mmol/l	4.82 (4.1–5.71)
TGL in mmol/l	1.6 (1.23–2.2)
LDLC > 2.6 mmol/l	259 (60.9)
TC > 5 mmol/l	183 (43.1)
HDLC < 1 mmol/l	124 (29.2)
TGL > 1.7 mmol/l	179 (41.2)
TGL ≥ 5.7 mmol/l	5 (1.2)
Non HDLC < 3.4 mmol/l	167 (39.3)
TC/HDLC ratio < 4.5 mmol/l	235 (55.3)
All normal LDLC, TGL, TC and HDLC concentrations	5 (12)
SBP, mmHg	139 (124–155)
DBP, mmHg	80 (73–91)

*DM* diabetes mellitus, *HT* hypertension, *FH* family history, *SU* sulphonylureas, *Met* metformin, *TZD* thiazolididiones, *BMI* body mass index, *HbA1c* glycated haemoglobin, *LDLC* low density lipoprotein cholesterol, *HDLC* high density lipoprotein cholesterol, *TC* total cholesterol, *TGL* triglycerides, *SBP* systolic blood pressure, *DBP* diastolic blood pressure, *IQR* inter quartile range

### Burden, pattern, management patterns of dyslipidaemia

Dyslipidaemia was documented in 374 study participants, accounting for 88%. Elevated LDLC concentrations was the commonest single lipid abnormality (60.9%) followed by elevated TC (43.1%), TGL (42.1%), non HDLC (39.3%) and low HDLC concentrations (29.2%). Severe hypertriglyceridemia defined as TGL levels ≥ 5.7 mmol/l was noted in only 4 (1%) study participants. Few patients were on lipid lowering drugs (LLD) i.e. statins with or without fibrates (20.9%) (summarised in Table 1).

### Socio-demographic, clinical and laboratory characteristics of the study participants at bivariate analysis

The variables that were statistically associated with the elevated lipid parameters of interest are shown in italics in Tables 2, 3 and Additional file 1: Table S1 and Additional file 2: Table S2. Additional file 1: Table S1 is uploaded as an additional file.

**Table 2:** Suboptimal LDLC concentrations in relation to socio-demographic and clinical characteristics at bivariable analysis

Characteristic	LDLC > 2.6 mmol/l	LDLC ≤ 2.6 mmol/l	OR 95% CI	p value
Age, median (IQR)	55.5 (48–67)	53 (43–62)	1.01 (0.99–1.02)	0.224
Gender				
*Male*	*71 (51.45)*	*67 (49.55)*	*1*	*0.001*
*Female*	*188 (68.12)*	*88 (31.88)*	*2.02 (1.33*–*3.07)*	
Type of hospital				
*Government*	*133 (66.83)*	*66 (33.17)*	*1*	*0.084*
*Private*	*126 (58.60)*	*89 (41.40)*	*0.76 (0.47*–*1.05)*	
Place of residence				
Rural	85 (62.96)	50 (37.04)	1	0.906
Urban	174 (62.37)	105 (37.63)	0.97 (0.64–1.49)	
Smoking				
*Smoker*	*8 (88.89)*	*1 (11.11)*	*1*	*0.135*
*Non smoker*	*251 (61.98)*	*154 (38.02)*	*0.20 (0.03–1.64)*	
Occupation				
Employed	132 (64.08)	74 (35.92)	1	0.526
Unemployed	127 (61.06)	81 (38.94)	0.88 (0.59–1.31)	
Co-existing HT				
*Yes*	*191 (67.25)*	*93 (32.75)*	*1*	*0.004*
*No*	*68 (52.31)*	*62 (47.69)*	*0.53 (0.35–0.82)*	
DM type				
*Type 1 DM*	*21 (38.18)*	*34 (61.82)*	*1*	*<0.005*
*Type 2 DM*	*234 (66.10)*	*120 (33.90)*	*3.16 (1.76–5.68)*	
Family history of DM				
*Yes*	*170 (66.15)*	*87 (33.85)*	*1*	*0.054*
*No*	*89 (56.69)*	*68 (43.31)*	*0.67 (0.45–1.01)*	
HIV co-morbidity				
Yes	10 (58.82)	7 (41.18)	1	0.745
No	249 (62.72)	148 (37.28)	1.18 (0.44–3.16)	
Median (IQR) age at diagnosis	53.5 (49–58)	46 (37–55)	1.01 (0.99–1.02)	0.379
*Median (IQR) years duration with DM.*	*3.5 (1–14)*	*4.5 (2–10)*	*1.02 (0.99–1.06)*	*0.165*
*BMI in kg/m* ^*2*^ *median (IQR)*	*28.7 (25–34.3)*	*27 (23–30.6)*	*1.04 (1.00–1.07)*	*0.041*
BP in mmHg, median (IQR)				
*SBP*	*130 (20–150)*	*139 (24–156)*	*1.01 (1.00–1.02)*	*0.015*
*DBP*	*70 (70–78)*	*80 (74–91)*	*1.02 (1.01–1.04)*	*0.002*
HbA1c (%), median (IQR)	8.95 (6.8–10.1)	9 (6.9–12.4)	0.98 (0.93–1.03)	0.488
Drugs				
*Insulin therapy*	*106 (58.89)*	*74 (41.11)*	*1*	*0.113*
*On OHA*	*151 (66.52)*	*76 (33.48)*	*1.39 (0.92–2.08)*	
Statin therapy n (%)				
*No*	*199 (60.86)*	*128 (39.14)*	*1*	*0.166*
*Yes*	*60 (68.97)*	*27 (31.03)*	*0.70 (0.42–1.16)*	

*DM* diabetes mellitus, *HT* hypertension, *FH* family history, *OHA* oral hypoglycaemic agents, *BMI* body mass index, *HbA1c* glycated haemoglobin, *SBP* systolic blood pressure, *DBP* diastolic blood pressure

**Table 3:** Suboptimal non HDLC concentrations in relation to socio-demographic and clinical characteristics at bivariable analysis

Characteristic	Non HDLC ≥ 3.4 mmol/l	Non HDLC < 3.4 mmol/l	OR 95% CI	p value
Age, median (IQR)	*56 (48–67)*	*53 (43–61)*	*1.02 (1.01–1.04)*	*0.001*
Gender				
* Male*	*65 (47.10)*	*73 (52.90)*	*1*	*<0.005*
* Female*	*181 (65.82)*	*94 (34.18)*	*2.16 (1.43–3.28)*	
Type of hospital				
* Government*	*126 (63.32)*	*73 (36.68)*	*1*	*0.134*
* Private*	*120 (56.07)*	*94 (43.93)*	*0.74 (0.50–1.10)*	
Place of residence				
Rural	85 (63.43)	49 (36.57)	1	0.267
Urban	161 (57.71)	118 (42.29)	0.79 (0.51–1.20)	
Smoking				
Smoker	6 (66.67)	3 (33.33)	1	0.662
Non smoker	240 (59.41)	167 (40.44)	0.73 (0.18–2.97)	
Occupation				
Employed	122 (59.22)	84 (40.78)	1	0.888
Unemployed	124 (59.90)	83 (40.10)	1.03 (0.69–1.52)	
Co-existing HT				
* Yes*	*179 (63.25)*	*104 (36.75)*	*1*	*0.025*
* No*	*67 (51.54)*	*63 (48.46)*	*0.62 (0.41–0.94)*	
DM type				
* Type 1 DM*	*16 (29.09)*	*39 (70.91)*	*1*	*<0.005*
* Type 2 DM*	*227 (64.31)*	*126 (35.69)*	*4.39 (2.36–8.17)*	
Family history of DM				
* Yes*	*162 (63.04)*	*95 (36.96)*	*1*	*0.066*
* No*	*84 (53.85)*	*72 (46.15)*	*0.68 (0.46–1.02)*	
HIV co-morbidity				
Yes	9 (52.94)	8 (47.06)	1	0.571
No	237 (59.85)	159 (40.15)	1.32 (0.50–3.51)	
*Median age at diagnosis*	*53 (48–58)*	*46 (37–55)*	*1.03 (1.01–1.04)*	*0.001*
Median (IQR) years duration with DM.	6 (1–15)	4 (2–10)	1.02 (0.99–1.05)	0.265
*BMI in kg/m* ^*2*^ *median (IQR)*	*28.7 (25–34.3)*	*27 (23–30.6)*	*1.07 (1.03–1.11)*	*<0.005*
BP in mmHg, median (IQR)				
* SBP*	*130 (120–150)*	*139 (124–156)*	*1.01 (1.00–1.02)*	*0.020*
* DBP*	*70 (70–78)*	*80 (74–91)*	*1.02 (1.01–1.03)*	*0.008*
HbA1c (%)	9.2 (6.8–10.1)	9 (6.85–12.4)	1.00 (0.95–1.05)	0.967
Drugs				
*I nsulin therapy*	*94 (52.22)*	*86 (47.78)*	*1*	*0.005*
* On OHA*	*149 (65.93)*	*77 (34.07)*	*1.77 (1.18–2.65)*	
On statin therapy n (%)				
* Yes*	*184 (56.44)*	*142 (43.56)*	*1*	*0.013*
* No*	*62 (71.26)*	*25 (28.74)*	*0.52 (0.31–0.87)*	

*DM* diabetes mellitus, *HT* hypertension, *FH* family history, *OHA* oral hypoglycaemic agents, *BMI* body mass index, *HbA1c* glycated haemoglobin, *SBP* systolic blood pressure, *DBP* diastolic blood pressure

### Independent predictors of elevated LDLC, TGL and non HDLC concentrations at multivariate analysis

The following identified independent predictors were indentified after logistic regression:

- Female gender (AOR 2.33 95% CI 1.43–3.80, p = 0.001), study site or private hospitals (AOR 0.54 95% CI 0.32–0.89, p = 0.017), type 2 DM (AOR 4.76 95% CI 2.03–11.14, p < 0.005), use of statin therapy (AOR 0.46 95% CI 0.24–0.90, p = 0.022) and diastolic BP (AOR 1.03 95% CI 1.01–1.05, p = 0.014) for elevated LDLC concentrations.
- Study site or private hospitals (AOR 0.59 95% CI 0.37–0.96, p = 0.032) and increased BMI (AOR 1.06 95% CI 1.02–1.10, p = 0.002) for elevated TGL concentrations.
- Female gender (AOR 2.20 95% CI 1.37–3.53, p = 0.001), study site or private hospitals (AOR 0.48 95% CI 0.29–0.79, p = 0.004), type 2 DM (AOR 3.13 95% CI 1.53–6.40, p = 0.002) and use of statin therapy (AOR 0.43 95% CI 0.23–0.80, p = 0.008) for elevated non HDLC concentrations (summarised as Additional file 1: Table S2 which is uploaded as an additional file).

## Discussion

This cross sectional study shows that dyslipidaemia was prevalent in the majority of the surveyed adult diabetic population. The rate of use of LLD was also low. The documented pattern of dyslipidaemia is consistent with what is described as diabetic dyslipidaemia [3].

Dyslipidaemia has been documented to be highly prevalent in African diabetic patients in most studies [6, 9–14]. Despite this high prevalence, varied patterns of dyslipidaemia have been described among African diabetic patients. A study done in a university referral hospital in Southern Ethiopia among 295 diabetics reported low HDLC concentration to be the most prevalent lipid abnormality (87.8%), followed by increased LDLC concentrations (63.7%), increased TC (34.6%) and increased TGL (29.8%) [9]. A similar pattern of dyslipidaemia was also noted in a small South African urban study of 150 adult diabetic patients (low HDLC-60.7%, increased LDLC-49.3%, increased TGL-45.3% and increased TC-29.3%) [11]. Results from the diabetes care study in Nigeria (Diabcare Nigeria study) in 531 diabetic patients reported low HDLC (76.3%) and increased TGL (60.7%) as the predominant lipid abnormalities [12]. The largest study assessing quality of diabetes care in 6 sub Saharan African countries (Diabcare Africa study) reported suboptimal TC and HDL concentrations in 36.2 and 39.4% of the study participants respectively. No study participant had elevated TGL concentrations despite the high prevalence of suboptimal glycaemic control (71% having HbA1c ≥ 6.5%) [13].

In our study, increased LDLC concentrations was the most prevalent, followed by elevated TC, TGL and low HDLC concentrations. Severe TGL defined as concentrations ≥ 5.7 mmol/l were uncommon in our study population.

Several reasons could explain the high prevalence of dyslipidaemia reported in our study and other similar African studies. Low rates of screening for dyslipidaemia and use of LLD have been noted in the majority of the sub Saharan African countries, possibly due to knowledge gaps among clinicians, low access to LLD and prohibitive costs of LLD and lipid profile testing. Two retrospective chart based studies done in outpatient diabetic clinics in Uganda [6] and South Africa [14] reported only 14 and 26% of the study participants having ever done a lipid profile assessment at least once in the previous 12 months and only 20.4 and 26.2% of the study participants respectively were receiving LLD. The Diabcare Africa study reported that about 45% of the study participants had ever performed a lipid profile assessment at least once in the past 1 year and only 13% were on LLD [13]. The LLD were reported to be unaffordable by similar studies performed in Cameroon [15] and in Benin, Sudan and Eriteria [16] reported LLD.

### Predictors of abnormal LDLC, TGL and non HDLC concentrations

Female gender, having type 2 DM, increased BMI and diastolic BP increased the likelihood of having abnormal LDLC, TGL and non HDLC concentrations while the use of LLD and receiving diabetes care from a private hospital reduced the likelihood.

An increased rate of dyslipidaemia among female diabetic patients has also been reported by studies performed in Ethiopia [9] and Botswana [10]. Compelling evidence suggests that dyslipidaemia is a common metabolic abnormality in type 2 DM compared to type 1 DM and in obese or overweight patients. Increased diastolic BP or hypertension and type 2 DM is part of the intimate cluster of metabolic disorders in metabolic or insulin resistance syndrome [3].

Unequivocal evidence supports the use of lifestyle modification and LLD in the management of dyslipidaemia among adult diabetic patients [3].

### Conclusions and recommendations

Dyslipidaemia is frequent among these adult diabetic patients in Uganda. The frequency of use of LLD was low. Due to this documented high prevalence, proactive screening for dyslipidaemia among adult diabetic patients should be encouraged. In addition to encouraging lifestyle measures, it is imperative that ready access to affordable lipid lowering drugs for optimal management of dyslipidaemia is improved in Uganda.

## Limitations

We cannot generalise these findings to the entire adult diabetic population in Uganda because the study was only done in urban tertiary health centres.

## Additional files

**Additional file 1.** Suboptimal TGL concentrations in relation to socio-demographic and clinical characteristics at bivariable analysis.

**Additional file 2.** Independent predictors of elevated LDLC, TGL and non HDLC concentrations at multivariable analysis.

## Abbreviations

DM: diabetes mellitus
HT: hypertension
LDLC: low dense lipoprotein cholesterol
HDLC: high dense lipoprotein cholesterol
TGL: triglycerides
CVD: cardiovascular diseases
T2DM: type 2 diabetes mellitus
BP: blood pressure
HbA1c: glycated haemoglobin
BMI: body mass index
LLD: lipid lowering drugs
IQR: interquartile range
