# Supplementary material for: Dyslipidaemia in a Black African diabetic population: burden, pattern and predictors
Source: BMC Res Notes. 2017 Nov 9;10:587. doi: 10.1186/s13104-017-2916-y (PMC5679328; doi:10.1186/s13104-017-2916-y)
Supplement: Supplementary file 1 — Additional file 1. Suboptimal TGL concentrations in relation to socio-demographic and clinical characteristics at bivariable analysis. [file 13104_2017_2916_MOESM1_ESM.docx]

**ADDITIONAL FILES**

**Table S1 Suboptimal TGL concentrations in relation to socio-demographic and clinical characteristics at bivariable analysis**

| **Characteristic** | **TGL>1.7 mmol/l** | **TGL ≤ 1.7 mmol/l** | **OR 95% CI** | **P value** |
| --- | --- | --- | --- | --- |
| Age, Median (IQR) | 55.5 (48-67) | 53 (43-62) | 1.01 (0.99-1.02) | 0.359 |
| **Gender**  **Male**  **Female** | **52 (37.68)**  **127 (46.01)** | **86 (62.32)**  **149 (53.99)** | **1**  **1.41 (0.93-2.14)** | **0.107** |
| **Type of hospital**  **Government**  **Private** | **94 (47.24)**  **85 (39.53)** | **105 (52.76)**  **130 (60.47)** | **1**  **0.73 (0.49-1.08)** | **0.114** |
| Place of residence  Rural  Urban | 61 (45.19)  118 (42.29) | 74 (54.81)  161 (57.71) | 1  0.89 (0.59-1.34) | 0.578 |
| Smoking  Non smoker  Smoker | 174 (42.96)  5 (55.56) | 231 (57.04)  4 (44.44) | 1  0.60 (0.16-2.28) | 0.455 |
| Occupation  Unemployed Employed | 91 (43.75)  88 (42.72) | 117 (56.25)  118 (57.28) | 1  1.04 (0.71-1.54) | 0.832 |
| Co-existing HT  No  Yes | 55 (42.31)  124 (43.66) | 75 (57.69)  160 (56.34) | 1  0.95 (0.62-1.45) | 0.796 |
| **DM type**  **Type 1 DM**  **Type 2 DM** | **19 (34.55)**  **159 (44.92)** | **36 (65.45)**  **195 (55.08)** | **1**  **1.54 (0.85-2.80)** | **0.151** |
| Family History of DM  No  Yes | 68 (43.31)  111 (43.19) | 89 (56.69)  146 (56.81) | 1  1.00 (0.67-1.50) | 0.981 |
| HIV co-morbidity  Yes  No | 9 (52.94)  170 (42.82) | 8 (47.06)  227 (57.18) | 1  0.67 (0.25-1.76) | 0.412 |
| Median (IQR) age at diagnosis | 53.5 (49-58) | 46 (37-55) | 1.01 (0.99-1.02) | 0.242 |
| Median (IQR) years duration with DM. | 3.5 (1-14) | 4.5 (2-10) | 0.98 (0.95-1.02) | 0.341 |
| **BMI in kg/m2 median (IQR)** | **28.7 (25-34.3)** | **27 (23-30.6)** | **1.07 (1.03-1.11)** | **<0.005** |
| **BP in mmHg, median (IQR)**  **SBP**  **DBP** | **130 (120-150)**  **70 (70-78)** | **139 (124-156)**  **80 (74-91)** | **1.01 (1.00-1.02)**  **1.02 (1.00-1.03)** | **0.049**  **0.017** |
| HbA1c (%) | 8.95 (6.8-10.1) | 9 (6.9-12.4) | 0.99 (0.94-1.04) | 0.582 |
| Drugs  Insulin therapy  On OHA | 72 (40)  105 (46.26) | 108 (60)  122 (56.51) | 1  1.29 (0.87-1.92) | 0.206 |
| **On statin therapy n (%)**  **No**  **Yes** | **136 (41.59)**  **43 (49.43)** | **191 (58.41)**  **44 (50.57)** | **1**  **0.73 (0.45-1.17)** | **0.191** |

DM-Diabetes Mellitus, HT-Hypertension, FH-Family history, OHA-oral hypoglycaemic agents, BMI-Body mass index, HbA1c-Glycated haemoglobin, SBP-Systolic blood pressure, DBP-Diastolic blood pressure, IQR-Inter quartile range.
