# Supplementary material for: Dyslipidaemia in a Black African diabetic population: burden, pattern and predictors
Source: BMC Res Notes. 2017 Nov 9;10:587. doi: 10.1186/s13104-017-2916-y (PMC5679328; doi:10.1186/s13104-017-2916-y)
Supplement: Supplementary file 2 — Additional file 2. Independent predictors of elevated LDLC, TGL and non HDLC concentrations at multivariable analysis. [file 13104_2017_2916_MOESM2_ESM.docx]

**Table S2 Independent predictors of elevated LDLC, TGL and non HDLC concentrations at multivariable analysis**

| **Elevated LDLC concentrations** | | | | |
| --- | --- | --- | --- | --- |
|  | **Unadjusted analysis** | | **Adjusted analysis** | |
| **Characteristic** | **OR (95% CI)** | **P-value** | **AOR (95% CI)** | **P-value** |
| Female gender | 2.02 (1.33-3.07) | 0.001 | 2.33 (1.43-3.80) | 0.001 |
| Study site: private hospital | 0.76 (0.47-1.05) | 0.084 | 0.54 (0.32-0.89) | 0.017 |
| Type of DM | 3.16 (1.76-5.68) | <0.005 | 4.76 (2.03-11.14) | <0.005 |
| Use of statin therapy | 0.70 (0.42-1.16) | 0.166 | 0.46 (0.24-.90) | 0.022 |
| DBP | 1.02 (1.01-1.04) | 0.002 | 1.03 (1.01-1.05) | 0.014 |
| **Elevated TGL concentrations** | | | | |
| Study site: private hospital | 0.73 (0.49-1.08) | 0.114 | 0.59 (0.37-0.96) | 0.032 |
| BMI in kgm2 | 1.07 (1.03-1.11) | <0.005 | 1.06 (1.02-1.10) | 0.002 |
| **Elevated non HDLC concentrations** | | | | |
| Female gender | 2.16 (1.43-3.28) | <0.005 | 2.20 (1.37-3.53) | 0.001 |
| Study site: private hospital | 0.74 (0.50-1.10) | 0.134 | 0.48 (0.29-0.79) | 0.004 |
| Type 2 DM | 4.39 (2.36-8.17) | <0.005 | 3.13 (1.53-6.40) | 0.002 |
| Use of statin therapy | 0.52 (0.31-0.87) | 0.013 | 0.43 (0.23-0.80) | 0.008 |

DM Diabetes mellitus, DBP diastolic blood pressure, BMI body mass index, AOR Adjusted Odds ratio
